# Antiparasitic triterpenic ester and ursolic acid lipid nanocapsule design, cytotoxicity, and permeability evaluation for oral delivery

**DOI:** 10.3389/fddev.2026.1846904

**Published:** 2026-06-03

**Authors:** Laura Schioppa, Cecilia Bohns Michalowski, Lucia Mamede, Romano Terrasi, Marie-France Hérent, Giulio G. Muccioli, Raphaël Frédérick, Michel Frederich, Ana Beloqui, Joelle Quetin-Leclercq

**Affiliations:** 1 Pharmacognosy Research Group, Louvain Drug Research Institute (LDRI), Université Catholique de Louvain (UCLouvain), Brussels, Belgium; 2 Department of Advanced Drug Delivery and Biomaterials, Louvain Drug Research Institute (LDRI), Université Catholique de Louvain (UCLouvain), Brussels, Belgium; 3 Laboratory of Pharmacognosy, Center of Interdisciplinary Research on Medicines (CIRM), University of Liège, Liège, Belgium; 4 Bioanalysis and Pharmacology of Bioactive Lipids, Louvain Drug Research Institute (LDRI), Université Catholique de Louvain (UCLouvain), Brussels, Belgium; 5 Medicinal Chemistry Group, Louvain Drug Research Institute (LDRI), Université Catholique de Louvain (UCLouvain), Brussels, Belgium

**Keywords:** antiparasitic diseases, Caco-2, cytotoxicity, lipid nanoparticle, oral delivery, permeability, plasmodium, triterpenes

## Abstract

**Introduction:**

Ursolic acid (UA), the antimalarial triterpenic mixture 8TTE (containing C-27 feruloyl and coumaroyl esters of ursane and oleane skeletons), and the semi-synthetic antitrypanosomal derivative ursolic acid O-phenyl propionate (UAOPP) exhibit high lipophilicity, which may limit their oral bioavailability. This study aimed to develop lipid nanocapsules (LNCs) to improve the solubility, intestinal permeability, and antiparasitic activity of these triterpenic compounds for potential oral delivery.

**Methods:**

LNCs formulations containing UA, 8TTE, and UAOPP were prepared and evaluated. A sensitive UPLC\x{2013}MS method was developed and validated for selected triterpenes quantification during transport studies across Caco-2 cell monolayers [limit of detection (LOD): 2 nM; limit of quantification (LOQ): 25 nM]. Cytotoxicity and permeability studies were conducted on Caco-2 cells to assess formulation safety and intestinal transport. *In vitro* antiparasitic activity of free and formulated compounds was evaluated against *Plasmodium falciparum* and *Trypanosoma brucei brucei* (Tbb).

**Results and discussion:**

The formulations were non-toxic to Caco-2 cells at concentrations up to 2 mg/mL. Permeability studies demonstrated enhanced transport for the formulated triterpenic esters, with permeability increases of up to 2.68-fold, shifting their classification from poorly absorbed to moderately absorbed compounds in humans [apparent permeability coefficient (Papp) > 1 × 10^–6^ cm/s]. Free UA showed the highest Papp value (4.95 × 10^–6^ cm/s ± 1.29 × 10^–7^), but caused epithelial integrity disruption after 2 h of incubation and during the following 48 h, whereas formulated UA induced minimal integrity loss at the same concentration. In antiparasitic assays, blank LNCs exhibited maximum non-toxic concentrations of 165 μg/mL against *P. falciparum* and 65 μg/mL against Tbb. At these maximum concentrations, formulated UA and 8TTE showed enhanced antiplasmodial activity; however, blank LNCs produced comparable effects. In contrast, UAOPP-loaded LNCs showed significantly improved antitrypanosomal activity by approximately 20% at 2.15 μM (cell viability: 38.20% ± 5.41) compared with free UAOPP (20.38% ± 8.80). These findings suggest that LNCs represent promising oral delivery systems for lipophilic triterpenes. Further in vivo pharmacokinetic and efficacy studies are needed to confirm their therapeutic potential.

## 1 Introduction

**FIGURE 1:**
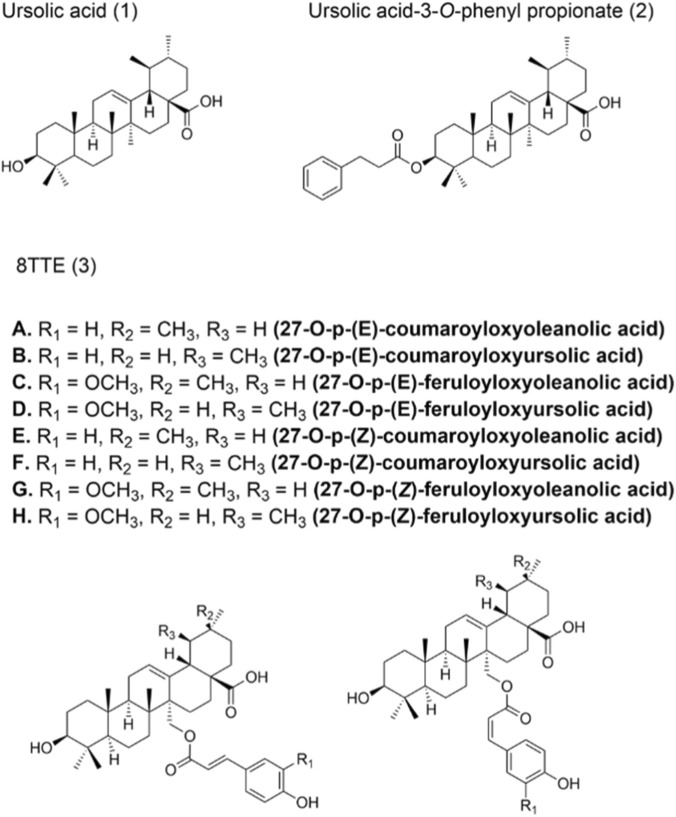
Structures of the studied triterpenic esters and ursolic acid.

African trypanosomiasis is a vector-borne disease caused by the *Trypanosoma brucei* (*T.b.*), a parasitic protist that is transmitted through the bite of a tsetse fly. The *T.b.* species is divided into three subspecies: *T.b. gambiense*, accounting for an often-chronic form of human African trypanosomiasis (HAT) in Central and Western Africa; *T.b. rhodesiense*, responsible for acute HAT in Eastern and Southern Africa; and *T.b. brucei*, which infects domestic and wild animals. *T.b. gambiense* and *rhodesiense* account for 98% and 2% of reported trypanosomiasis cases, respectively. In 2019, less than 1,000 cases of HAT have been reported, with approximately 2.5 million people screened per year [World Health Organization (WHO), 2025]. HAT or sleeping sickness is endemic in 36 countries in sub-Saharan Africa, so its incidence is probably underestimated. Despite the latest improvements and a decrease in cases, millions of people remain at risk, with treatment toxicity and parasite resistance threatening current therapies. Furthermore, resources of the affected zones are often scarce, particularly in remote areas where the disease is mostly found, urgently calling for safer and easier therapies (Gao et al., 2020; Hollingshead and Bermudez, 2024). Medicinal chemistry approaches to synthetize effective but less cytotoxic or more stable derivatives of the antiparasitic ursolic acid (UA) and oleanolic acid were explored, leading to the hemi-synthesis of the lead compound ursolic acid O-phenyl propionate (UAOPP) (Gao et al., 2020) (Figure 1). This C-3 ester of ursolic acid [World Health Organization (WHO), 2025] was found to be active in the micromolar range on *Trypanosoma brucei brucei* (*Tbb*) (3.5 µM) with high selectivity (SI > 40.7 on the WI38 cell line) and showed significant parasitemia inhibition (61.2% ± 27.8%) on day 5 post-infection in *Tbb*-infected mice at 50 mg/kg (Schioppa et al., 2021a). Notably, metabolomics studies have confirmed the therapeutic potential of these molecules, suggesting that they may act by targeting amino acid degradation pathways (Mamede et al., 2025). Malaria is a parasitic infection caused by the bite of the vector of an infective female Anopheles mosquito. Among the five *Plasmodium* species that infect humans, *P. falciparum* is responsible for the most severe cases of malaria, and if left untreated, it can be fatal. In 2023, the World Health Organization (WHO) reported an estimated 263 million cases of malaria globally and 597,000 deaths. Most of these deaths occur in sub-Saharan Africa, where children under 5 years old are particularly vulnerable (Malaria, 2024). Considering the death and illness burden, the disease continues to be a great drain on many national economies (CDC, 2022). Many of the affected populations live in remote rural areas with limited access to adequate health services, which complicates disease control, diagnosis, and treatment, thereby increasing the need for effective treatment with an easy route of administration. Previous research in our laboratory showed the promising potential of the purified mixture of eight triterpenic esters, 8TTE (Hollingshead and Bermudez, 2024) (Figure 1), containing four feruloyl isomers (8Ef) and four coumaroyl isomers (8Ec) on ursane and oleanane skeletons. 8TTE is considered a potential hit (IC_50_ = 1.66 ± 0.54 μg/mL = 2.6 µM on 3D7, SI = 34.6) due to its *in vivo* (*Plasmodium berghei* infected mice) significant parasitemia inhibition (27.8% ± 5.4%) on day 4 post-infection at 50 mg/kg (p < 0.01) (Beaufay et al., 2017). They possess the same *in vitro* activity regardless of the substitution, and the *E* and *Z* isomers transform to each other after purification. That is why we decided to keep them as a mixture. Their unique antiplasmodial profile with activity onset in the early-ring stage of the parasite, the inhibition of aminopeptidase PfA-M17, and perturbations in parasite hemoglobin metabolism confirms their interest as antiplasmodial leads (Mamede et al., 2026).

Considering the high lipophilicity of triterpenes, their generally low permeability and poor solubility, and the limited information available on the studied pentacyclic triterpenes (PTs), our rational was to explore a formulation strategy able to efficiently encapsulate lipophilic compounds and capable to enhance their intestinal absorption. Among the many approaches available (e.g., solid dispersion and cyclodextrin complexation) (Soica et al., 2014), we selected lipid nanocapsules (LNCs), considering their lipidic inner core and our previous experience on the encapsulation of lipophilic drugs within these nanocarriers (Guilbaud et al., 2025). These nanocapsules contain an oily core composed of medium-chain triglycerides surrounded by a surfactant shell made of a PEGylated surfactant and, optionally, lecithin or other co-surfactants (Matoug et al., 2016). Numerous active ingredients, mainly drugs with lipophilic properties, have already been incorporated into LNCs, including fluticasone propionate, essential oil constituents (eugenol, carvacrol, and trans-cinnamaldehyde), paclitaxel, and ibuprofen (Valcourt et al., 2016; Lamprecht et al., 2004). The advantages of LNCs include small particle size (20–100 nm), good physical stability (over 18 months), and easy scale-up using a phase-inversion temperature (PIT) method, which is a low-energy, organic solvent-free process (Huynh et al., 2009). In this study, we represent the first exploration of lipid nanocapsules (LNCs) for enhancing the bioavailability of triterpenic esters as antiparasitic drugs. Although some studies have described UA nanoparticles, primarily aiming at improving its inherently poor water solubility, low intestinal absorption, and susceptibility to first-pass metabolism (as UA is classified as a BCS Class IV compound) (Qiu et al., 2019; Valdés et al., 2016; Kaushik et al., 2025), the application of nanoparticles to triterpenic esters, particularly for antiparasitic purposes, remains unexplored. Despite their promising antiparasitic activity, UA and related triterpenic esters are characterized by poor aqueous solubility, limited intestinal permeability, and low oral bioavailability, which significantly restrict their therapeutic potential. So far, no optimized nanocarrier system has been systematically evaluated for improving their oral delivery in the context of antiparasitic therapy. In this study, we hypothesize that encapsulation of UA and triterpenic esters into LNCs would enhance their solubility and intestinal permeability, thereby improving their suitability for oral administration. The objective of this study was, therefore, to design, characterize, and evaluate LNC formulations loaded with these compounds, with a focus on cytotoxicity and permeability performance as a first step toward improved oral antiparasitic delivery.

## 2 Materials and methods

### 2.1 Chemicals and material

Twigs of *Keetia leucantha* (K. Krause) Bridson (syn. *Lectronia leucantha* Krause, Rubiaceae) were collected in Benin (Adjarra, Ouémé) in July 2011 and August 2012 and identified at the National Botanic Garden of Belgium in Meise (compared to voucher number BR0000005087129). The 8TTE triterpenic mixture was obtained as described before, with a purity of >98% (Bero et al., 2013). UAOPP was hemi-synthetized as previously described from UA (Schioppa et al., 2021a), with a purity of >97%. All used organic solvents (VWR, Belgium) were of HPLC grade. Water was purified and deionized with a Milli-Q system manufactured by Millipore (Bedford, MA, United States). The estradiol valerate and UA (purity 99%) were purchased from Aca Pharma NV (Certa, Belgium), and estradiol valerate was used as the internal standard. Medium-chain triglycerides (caprylic/capric acid triglycerides) (Labrafac® WL 1349) and glyceryl monooleate (Peceol®) were kindly provided by Gattefossé (Saint-Priest, France). Soybean lecithin (Lipoid® S100) was purchased from Lipoid GmbH (Ludwigshafen, Germany). Sodium chloride (NaCl), macrogol 15 hydroxystearate (Kolliphor® HS15), suramin sodium salt (antitrypanosomal commercial drugs, purity >99%), 3-(4,5-dimethylthiazol-2-yl)-2,5-diphenyltetrazolium bromide (MTT), dimethyl sulfoxide (DMSO), and Triton-X 100 were bought from Sigma-Aldrich (St. Louis, MO, United States).

### 2.2 HPLC–PDA and UPLC–MS analyses

Samples were analyzed using a HPLC-PDA system according to the previously validated method (Schioppa et al., 2021b). For UPLC–MS analysis, an Acquity UPLC class H liquid chromatography system coupled to a Xevo TQ-S mass spectrometer was used. The column used was an ACQUITY UPLC® HSS C18 (1.8 µm, 2.1 × 100 mm) with a flow rate of 0.3 mL/min using a gradient solvent system: solvent A (20%) consisted of H_2_O + 0.1% formic acid; solvent B (45%) was acetonitrile, and solvent C (35%) was MeOH. The gradient was maintained for 4 min, then modified to reach solvents B (50%) and C (50%) at 6 min, followed by isocratic elution until 10 min. Starting conditions were restored from 11 min to 15 min. The injection volume was 2 μL; autosampler and column temperatures were set at 4 °C and 25 °C, respectively.

### 2.3 Method validation and preparation of standards and quality control (QC) samples

8TTE, UAOPP, UA, and IS stock solutions were prepared in methanol and diluted to have calibration standard solutions with concentrations of 25, 50, 100, 1,000, 2,500, and 5,000 nM. Working solutions for quality control (QC) samples at low, medium, and high levels with concentrations of 75, 1,500, and 3,750 nM, respectively, were prepared in the same manner. The analyte/IS peak ratio was determined using the UPLC–MS method specified above (n = 3, k = 2, mean ± SD). The UPLC method was developed from the validated method described by Schioppa et al. (2021b) and validated for these analyses. The UPLC method was validated with three independent series of experiments. The same mobile phase was used all along with one series. Response function, linearity, selectivity, precision, trueness, accuracy, limit of detection (LOD), limit of quantification (LOQ), and quantification range were investigated. The method selectivity was confirmed by the lack of the interference peak in blank at the retention times of the compounds of interest. The validation of the presented bioanalytical method agrees with EMA guidelines, with trueness and precision values lower than 15%, expressed as relative bias (RB) and the relative standard deviation (RSD), respectively (Medicines Agency, 2017). In addition, total error (sum of systematic and random error) was used as decision criteria for the validation process (Hubert et al., 2004; Hubert et al., 2007a; Hubert et al., 2007b; Hubert et al., 2008; Rozet et al., 2011; Lautié et al., 2012). Statistical analyzes were performed using JMP v12 software. The acceptance limits (λ) were set at ± 20%, as usually accepted (Lautié et al., 2012; Rafamantanana et al., 2009). The probability to obtain future results within these limits (β) was set at 95%.

### 2.4 Preparation of triterpenic ester- and ursolic acid-loaded lipid nanocapsules

**FIGURE 2:**
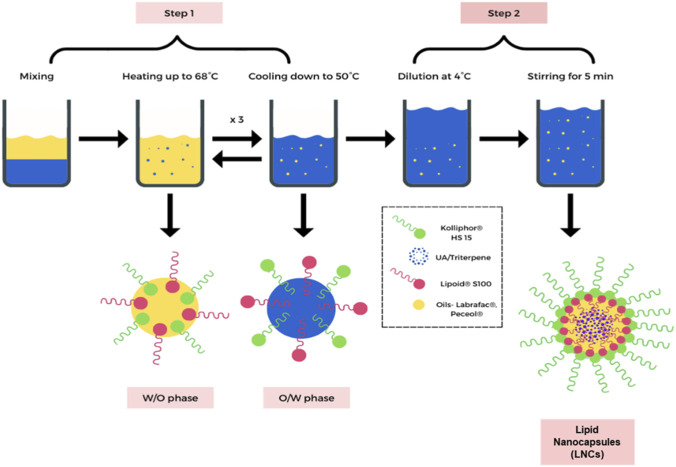
Phase-inversion temperature method for the preparation of lipid nanocapsules (LNCs).

LNCs were prepared following a PIT method, adapted from the approach described by Heurtault et al. (2002). In brief, soybean lecithin (Lipoid® S100, 6.7 mg), macrogol 15-hydroxystearate (Kolliphor® HS15, 60 mg), glyceryl monooleate (Peceol®, 51 mg), medium-chain triglycerides (Labrafac® WL 1349, 405 mg), and NaCl (26.5 mg) were combined under stirring (220 rpm) after addition of Milli-Q water (512.5 mL) and heated to 68 °C. The mixture was cooled to 50 °C, and three heating–cooling cycles (50 °C–68 °C) were applied. During the second cycle, reverse micelles containing the hydrophobic drug (5–6 mg) dissolved in 20 µL DMSO and Span 80 (40 mg) were incorporated at 64 °C to facilitate encapsulation of poorly water-soluble esters. The temperature was maintained within a controlled range (±1 °C) throughout the cycles to ensure reproducibility. In the final cycle, 1.25 mL of cold water (4 °C) was added at the specific phase-inversion point of 60.4 °C under high-speed stirring (350 rpm, 5 min) to fix the nanocapsule structure. Stirring speed and temperature points were kept constant across all batches to ensure batch-to-batch reproducibility. A scheme of the preparation is illustrated in Figure 2. The LNCs were filtered using a 0.45-μm filter and stored at 4 °C until use. Blank RM-LNCs were prepared following the same protocol in the absence of the triterpenic compound. All formulations were prepared in three independent batches (n = 3). The LNC composition was expressed as the ratio between the total mass of lipid excipients (medium-chain triglycerides, soybean lecithin, macrogol 15-hydroxystearate, and glyceryl monooleate) and the final formulation mass, in accordance with established formulations of lipid nanocapsule systems. A schematic representation of grafted LNC formulation developed for pentacyclic triterpenes and adapted from Eissa et al. (2015) method is available in Supplementary Material (Supplementary Figure 1S).

### 2.5 Characterization of LNCs

The LNC particle size and the polydispersity index (PDI) were characterized by dynamic light scattering (DLS) using a Zetasizer Nano ZS (Malvern Instruments Ltd., Worcestershire, United Kingdom). The zeta potential was measured by laser Doppler velocimetry (LDV) also using a Zetasizer Nano ZS (Malvern Instruments Ltd., Worcestershire, United Kingdom). Prior to analysis, the samples were diluted in Milli-Q water to avoid multiple scattering effects. Characterization was performed on three independent batches (n = 3), with each sample recorded in three technical replicates to ensure statistical robustness. Encapsulation efficiency (EE%) was determined by separating free (non-encapsulated) drug from LNC dispersions by centrifugation, followed by quantification of drug content using UPLC–MS analysis. EE% was calculated as follows:

$$EE\;\left(\%\right)=\frac{\text{total}\;\text{drug}\;\text{amount}-\text{free}\;\text{drug}\;\left(\text{non}\;\text{trapped}\right)}{total\;drug\;amount}\;x\;100.$$

Drug loading (%) was calculated based on the amount of encapsulated drug determined after separation, relative to the total mass of the formulation. Encapsulation efficiency (EE%) and drug loading (%) were used to characterize drug incorporation within LNCs. EE% reflects the fraction of drug entrapped within the lipid nanocapsules, whereas drug loading (%) describes the proportion of drug relative to the total formulation mass.

*In vitro* culture parameters

Bloodstream forms of *Tbb* (strain 427) were cultivated *in vitro* in HMI-9 medium (Gibco) supplemented with 10% heat-inactivated fetal bovine serum (FBS), 150 mM L-cysteine, and 20 mM beta-mercaptoethanol (Sigma-Aldrich) at 37 °C and 5% CO_2_. The asexual erythrocytic stage of *P. falciparum* (*Pf* 3D7, a chloroquine-sensitive strain originally isolated from a patient in The Netherlands) was cultivated *in vitro* in RPMI 1640 (Gibco) medium supplemented with 10% heat-inactivated human pooled serum (O), hypoxanthine (44 mg/L), glucose (1.76 g/L), and 1% of 10 mg/mL gentamycine solution, at 37 °C in an adapted atmosphere (5% CO_2_, 5% O_2_, and 90% N_2_). Caco-2 cells were cultured in medium consisting of Dulbecco’s modified Eagle’s minimal essential medium (DMEM) supplemented with 10% (v/v) heat-inactivated fetal bovine serum (HyClone®, Thermo Scientific, United Kingdom), 1% (v/v) L-glutamine, 1% (v/v) nonessential amino acids, and penicillin/streptomycin solution (10 units/10 μg/mL) and were incubated at 37 °C in a 10% CO_2_ humidified incubator. The medium was changed every 2 days. Dulbecco’s modified Eagle’s medium (DMEM)–GlutaMAX, penicillin–streptomycin (P/S), FBS, phosphate-buffered saline (PBS), and trypsin (0.25%)-containing ethylenediaminetetraacetic acid (EDTA, 0.02%) were purchased from Thermo Fisher Scientific (Invitrogen, United Kingdom).

### 2.6 Cytotoxicity on Caco-2 cells

The viability of Caco-2 cells against isolated compounds and blank and loaded LNC formulations was evaluated as described by Memvanga et al. (2013). In brief, Caco-2 cells were seeded on 96-well plates (2 × 10^4^ cells/well) for 24 h. Once the cells were confluent, they were washed with PBS at 37 °C and treated with 100 µL of free drugs, unloaded LNC, or loaded LNC dispersed in Hank’s salt balanced solution (HBSS). The samples were prepared at concentrations varying from 0.1 mM to 4 mM, with HBSS serving as a negative control, and 1% (v/v) Triton X-100 serving as a positive control. After 2 h of incubation, cells were washed with HBSS at 37 °C, treated with 100 µL of MTT solution (0.5 mg/mL in DMEM) and further incubated for 3 h. Next, 200 µL of DMSO was added to solubilize the formazan crystals formed during the incubation, and the product of the reaction was measured at 545 nm using a Multiskan Spectrum microplate reader (Thermo Fisher Scientific Inc., Waltham, MA, United States). Cell viability rates of the samples were calculated based on absorbance (Abs) as follows:

$$Cell\;viability\;\left(\%\right)=\frac{\text{Abs}\;\text{sample}}{Abs\;negative\;control\;cells}\;x\;100.$$

### 2.7 Antiparasitic activities

Trypanosomal viability was measured using the AlamarBlue assay. Samples stock solutions were first diluted at 0.2 mg/mL in fresh medium and then in eight serial three-fold dilutions (25 µL transferred in 75 µL) in 96-well microtiter plates. An aliquot of 50 μL of parasite culture (10^5^ or 5 × 10^4^ parasite/mL, respectively) was added to 50 µL of the tested sample solution (final concentration range: 100–0.05 μg/mL). After 72 h of incubation, 10 µL of reagent (Thermo Fischer Scientific, two-fold diluted in PBS) was added in each well and incubated for 4 h. The fluorescence of the reduced reagent associated with metabolically active parasites was read at 530 (excitation) and 590 (emission) nm. Suramin sodium salt was used as the positive control, with an initial concentration of 10 μg/mL. *Plasmodium* viability was determined indirectly by measuring the activity of the plasmodial enzyme lactate dehydrogenase (pLDH). Sample stock solutions were first diluted at 2 mg/mL in fresh medium and then in eight serial three-fold dilutions (12 µL transferred in 24 volumes of medium) in 96-well microtiter plates. An aliquot of 225 μL of parasite culture (2% parasitemia and 1% hematocrit) was added to the tested sample solution (final concentration range: 192–0.09 μg/mL). After 48 h, plates were frozen to lyse the red blood cell membrane. Then, after parasite membrane lysis with saponin and Triton X-100, pLDH activity was evaluated indirectly by measuring the formation of the reduced coenzyme reduced acetyl pyridine adenine dinucleotide (APADH). Indeed, in the presence of phenazine methosulfate (PES), it reduced nitro blue tetrazolium (NBT) in the formazan product, the absorbance of which was detected by spectrophotometry at 630 nm. A reference antiplasmodial drug, artemisinin (Sigma-Aldrich 98% purity), was used as the positive control, with a 0.1 mg/mL stock solution (tested concentration range: 100–0.05 ng/mL).

### 2.8 Transport studies

The *in vitro* transport studies were carried out as described by Memvanga et al. (2013). Caco-2 cells (5 × 10^5^ cells/well) were seeded on 12-well cell culture inserts, with a 1-µm pore diameter and 0.9-cm^2^ surface area (Corning Costar®, NY, United States), and were grown in culture medium at 37 °C in an atmosphere of 10% CO_2_. Cell culture medium was added to the apical (0.5 mL) and basolateral (1.2 mL) sides, and the medium was replaced every 2 days. After 21 days of incubation, only Caco-2 cell monolayers with initial transepithelial electrical resistance (TEER) values above 300 Ω/cm^2^ were selected. Before the transport study, the culture medium was replaced with pre-heated (37 °C) HBSS. After the cell monolayer was equilibrated for 30 min at 37 °C, TEER values of monolayers were determined in triplicate. The apical-to-basolateral transport experiments across Caco-2 cell monolayers were conducted by adding 0.5 mL of triterpenic esters or acid-free drugs or 0.5 mL of dispersed formulations in HBSS (at lowest cytotoxic concentration) on the apical side of the inserts and 1.2 mL of HBSS on the basolateral side. After 2 h, samples from the basolateral compartment were withdrawn to determine the permeation of free drug or loaded LNCs. Samples were then evaporated under gentle nitrogen stream, dissolved in methanol, and centrifuged at 6,500 rpm for 5 min, and the supernatants were filtered and injected into the UPLC–MS system. The amount of the drug that crossed the Caco-2 cell monolayers was determined using UPLC–MS as described before. The apparent permeability coefficient (P_app_) was determined using the following equation:

$$P_{app}=\frac{\text{dQ}}{dt}\;x\;\frac{\text{dQ}}{C_{0}A},$$

where dQ/dt (transport rate) is the amount of the drug (μg) appearing per time unit (s) in the receiver compartment, C_0_ is the initial concentration in the donor compartment (μg/mL), and A is the surface area of the monolayer (A = 0.9 cm^2^).

### 2.9 Data analysis

Analytical and regression parameters were calculated from calibration data using Microsoft Excel and GraphPad Prism (version 8.4.2). Unless otherwise stated, all biological experiments were performed in at least three independent replicates (n = 3), and the results are expressed as the mean ± standard deviation (SD). Comparative evaluations were performed using a two-way analysis of variance (ANOVA), followed by Tukey’s *post-hoc* test for multiple comparisons. Dunnett’s test was specifically employed to compare treatment groups against blank LNCs at equivalent concentrations. A *p*-value <0.05 was considered statistically significant. Unless otherwise stated, the data are expressed as the mean ± SD for n = 3 experiments. For mass spectrometry analysis, MassLynx software was used.

## 3 Results and discussion

### 3.1 Validation of the UPLC–MS method

Due to the carboxyl in the chemical structures of tested triterpenic esters and acid, signal intensity was higher in negative mode. After fragmentation in the collision cell, the most abundant and stable transition signals for each compound are tabulated in Table 1. UA produced only one fragment ion at both higher and lower collision energies; therefore, we could only use one transition. This was already observed previously by other researchers (Xia et al., 2011). 8TTE is a mixture of eight triterpenic esters, four isomers of coumaroyltriterpenic acid (8Ec), and four of feruloyltriterpenic acid (8Ef). The validated method is selective enough to make distinction among those isomers by molecular weights and transitions of both quantitative and qualitative fragments (see Table 1). The validated method is precise, accurate, specific, and selective for the intended purposes. Full-validation results can be found in Supplementary Material, including MRM chromatograms (Supplementary Material, 2.1), selectivity and specificity (Supplementary Material, 2.2), linearity and lower limits of quantitation and detection (Supplementary Material, 2.3), precision and accuracy (Supplementary Material, 2.4), uncertainty of measurement (Supplementary Material, 2.5), and stability results (Supplementary Material, 2.6).

**TABLE 1:** Mass spectrometry characteristics of tested compounds. A, UA; E, UAOPP; 8Ec, 8TTE coumaroyl; 8Ef, 8TTE feruloyl. IS (Internal Standard) = estradiol valerate.

Compound	Isomer	R.T.	Parent ion	Transitions (*m/z*)	CV (V)	CE (eV)
(A)	—	7.55	455.3600	407.2649	62	36
(E)	—	9.80	587.3600	148.8700	94	46
​	—	9.80	587.3600	437.3300	94	46
(8Ec)	CoumaroylOA	2.92	587.3600	162.9600	40	40
​	CoumaroylUA	3.35	617.4500	453.1200	40	40
(8Ef)	FeruloylOA	3.00	647.2700	192.7400	40	40
​	FeruloylUA	3.63	647.2700	453.0000	40	40
IS	—	5.75	355.1400	253.0700	8	32
​	—	5.68	355.1400	100.7700	8	32

CV, cone voltage (V); CE, collision energy (eV).

### 3.2 LNC formulation characterization

The phase inversion process was used to prepare LNCs. The mean particle sizes of the LNCs were below 220 nm ± 1.100 nm (Table 2). This nanometric size is critical for oral delivery as particles under 200–300 nm are more likely to bypass the intestinal mucus barrier and undergo efficient transcellular uptake by enterocytes or M-cells. The small PDI index (<0.15), when nanocapsules are drug-loaded, indicated the homogeneity of the LNC in terms of size distribution, suggesting that the PIT process remains robust even upon the incorporation of bulky triterpenic structures. Encapsulation efficacy was higher for UA and its hemi-synthetic ester (>94%) and lower for the natural esters’ mixture (60%). This disparity likely reflects differences in the lipophilicity and molecular packing within the LNC lipid core; although UA and UAOPP integrate efficiently into the triglyceride matrix, the structural diversity of the 8TTE natural mixture may lead to limited core capacity or the premature expulsion from the lipid core during the phase inversion. The negative charge of the zeta potential increased when LNCs were drug-loaded, suggesting a higher stability than the blank formulation, with values in accordance with those reported for other similar formulations (Xu et al., 2018). This suggests that the drug molecules are successfully sequestered within the lipid nanocapsules rather than being adsorbed onto the surface, where they might otherwise destabilize the surfactant shell through steric or electrostatic interference.

**TABLE 2:** Physicochemical characterization of the different lipid nanocapsules (mean ± SEM, n = 3). PDI, polydispersity index; EE, encapsulation efficacy.

LNCs	Particle size	PDI	Zeta potential	EE
	nm (Z avg)	Mw/Mn	mV	%
UA	219.8 ± 1.100	0.152 ± 0.018	−4.98 ± 0.143	96.70 ± 2.20
UAOPP	212.7 ± 3.402	0.136 ± 0.021	−5.43 ± 0.200	94.12 ± 1.04
8TTE	207.2 ± 2.757	0.090 ± 0.038	−4.71 ± 0.344	60.50 ± 0.61
Blank	221.2 ± 2.193	0.189 ± 0.022	−1.48 ± 0.026	—

### 3.3 Caco-2 cell viability study

**FIGURE 3:**
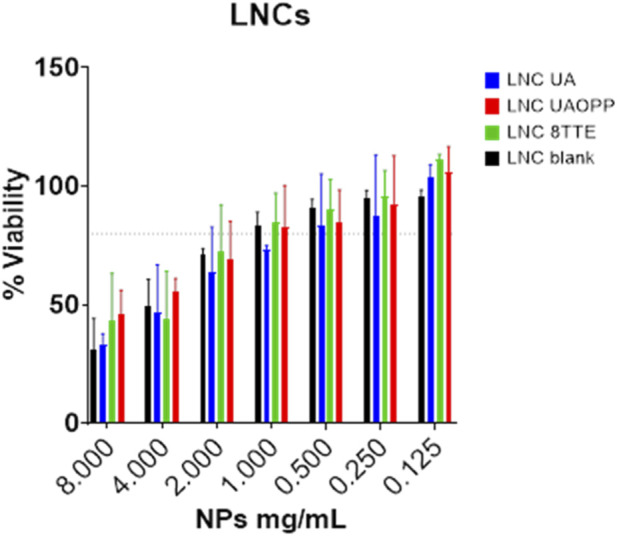
Cell viability of Caco-2 cells treated with LNCs of UA, UAOPP, and 8TTE at different concentrations (0.125 mg/mL to 8 mg/mL) (mean ± SD; N = 3, n = 3) using ANOVA followed by Dunnett’s test, compared to blank LNCs at the same concentration.

To assess the *in vitro* safety and cytocompatibility of the designed LNCs and free drugs, a cell viability study was performed using the MTT assay in Caco-2 cells (1 μM–100 µM free drugs and 8 mg/mL–0.125 mg/mL NPs for LNC). All tested free compounds showed cytotoxicity above 250 µM, with UA being the most cytotoxic (Supplementary Figure 3S). A similar UA cytotoxicity was reported by Qiang et al. (2011). As illustrated in Figure 3, the cytotoxicity of the LNCs was concentration-dependent. Both empty and drug-loaded LNCs exhibited reduced cell viability at concentrations exceeding 2 mg/mL, suggesting that the observed effect is primarily driven by the lipid nanocapsules concentration rather than the encapsulated active ingredient. According to these results, 2 mg/mL was chosen as the maximum NP concentration for the transport study across the Caco-2 monolayer. The observed cytotoxicity at higher LNC concentrations is likely attributable to the high density of surfactants required for the PIT method. Although PEGylated surfactants such as Kolliphor HS15 are generally recognized as safe, they can induce transient perturbations in the cell membrane at high concentrations to facilitate drug entry, which may lead to reduced viability in a static *in vitro* environment. However, it is important to note that the static conditions of the MTT assay do not account for the protective role of the intestinal mucus layer or the dynamic dilution effects occurring *in vivo*. Consequently, the 2 mg/mL threshold represents a conservative safety limit for the Caco-2 model in this study.

### 3.4 Transport study through the Caco-2 cell monolayers

**FIGURE 4:**
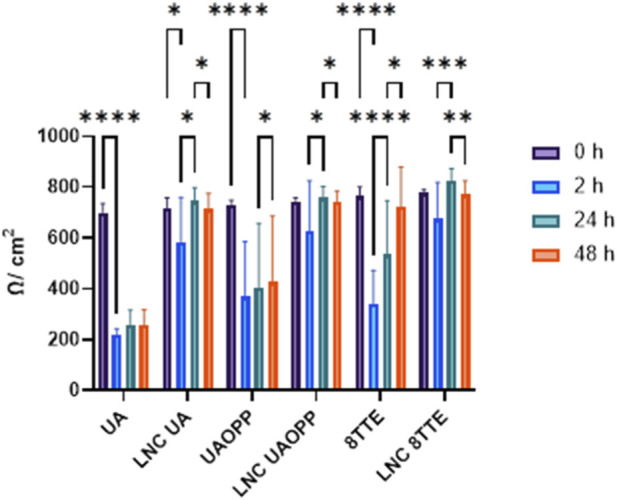
Transepithelial electrical resistance (TEER) of Caco-2 cells after administration of LNCs or free drug of UA, UAOPP, and 8TTE for 48 h (n = 3; mean ± SD). ANOVA analysis (Tukey’s multiple comparisons test): *p < 0.05 and ****p < 0.0001 compared before and after incubation (2 h) and with the previous time point (2 h vs. 24h; 24 h vs. 48 h).

Based on the described cytotoxicity, Caco-2 monolayers were incubated for 120 min at 37 °C with concentrations tabulated in Table 3 corresponding to the maximal non-cytotoxic NP concentration (2 mg/mL). TEER is usually used to monitor the tight junctions of the epithelium (Srinivasan et al., 2015). During the experiment, to evaluate the cell monolayer integrity after free or formulated molecule passage, TEER values were measured after 2h, 24h, and 48 h of treatment. As shown in Figure 4, a significant change in TEER values after 2 h of incubation of free drugs was observed (*p* < 0.05), in particular for UA. The lack of TEER recovery after 48 h indicates persistent monolayer disruption. Although MTT assays did not show marked cytotoxicity, differences in experimental conditions between viability assays and confluent monolayers limit direct comparison. Thus, the increased permeability observed at higher UA concentrations is likely an artifact of the loss of monolayer integrity rather than a result of enhanced physiological transport. Both 8TTE and UAOPP showed a decrease in TEER after treatment, but almost twice less than UA, keeping the TEER value higher than 300 Ω/cm^2^, which is the minimum accepted value for considering cellular barrier integrity (Hubatsch et al., 2007). Furthermore, 8TTE showed TEER recovering over time after the experiment (p < 0.05). All loaded LNCs showed a slight decrease in TEER values after 2 h of incubation (only significant for LNC UA), followed by full recovery within 24 h (Figure 4), indicating intact epithelial tight junctions for all formulations (Schulzke and Fromm, 2009). This transient and reversible reduction in TEER suggests that LNCs interact with the epithelium in a non-destructive manner, likely through localized and temporary membrane fluidization. The apical-to-basolateral transport permeability (P_app_ values) of the LNC formulations decreased in the following order: LNC 8TTE > LNC UAOPP > LNC UA (Table 3). The quantity of the drug transported across the Caco-2 monolayers ranged between 0.16%–3.14% (0.05–1 µM) and 0.44%–1.44% (0.04–0.3 µM) of the respective free drugs and loaded LNCs in the donor compartment. The P_app_ values of LNCs from LNC UAOPP and LNC 8TTE were 1.87- and 2.68-fold higher than those for the free drug, respectively, showing that the P_app_ value of triterpenic esters could be enhanced using LNC formulations.

**TABLE 3:** *In vitro* transport across Caco-2 parameters and permeability data.

Compound	µM_th_	µM_exp_	Recovery %	P_app_	Δ P_app_
UA	32	33.15	100.39 ± 5.03	4.95E − 06 ± 1.29E − 07	​
LNC UA	32	31.13	100.60 ± 12.48	4.95E − 07 ± 1.03E − 08	0.1
UAOPP	66	68.50	94.33 ± 16.00	5.35E − 07 ± 1.50E − 08	​
LNC UAOPP	66	67.65	96.64 ± 12.48	1.00E − 06 ± 7.14E − 08	1.87
8TTEc	14	14.23	70.20 ± 8.76	2.93E − 07 ± 2.34×E − 08	​
LNC 8TTEc	14	15.46	92.25 ± 2.83	8.19E − 07 ± 4.69E − 08	2.79
8TTEf	14	16.16	98.13 ± 5.87	1.00E − 06 ± 2.58E − 08	​
LNC 8TTEf	14	15.46	96.18 ± 3.15	2.56E − 06 ± 2.92E − 07	2.56
8TTE	14	15.19	84.16 ± 7.27	6.47E − 07 ± 0.6E − 08	​
LNC 8TTE	14	15.46	94.21 ± 7.17	1.69E − 06 ± 2.52E − 07	2.68

Δ P_app_ compares free and loaded LNCs for the same compound at the same concentration. 8TTEc, 8TTE coumaroyl, 8TTEf, 8TTE feruloyl, 8TTE, average of 8TTE coumarate and ferulate. All concentrations are expressed in micromolar (µM). Values followed by ‘th’ represent theoretical concentrations, while ‘exp’ denotes experimentally determined concentrations.

In the case of LNC UA, encapsulation of UA within LNCs did not result in a significant enhancement of permeability. The P_app_ value of LNC UA (4.95 × 10^−7^ ± 1.03 × 10^−8^ cm/s) was lower than that of free UA (4.95 × 10^−6^ ± 1.29 × 10^−7^ cm/s. This reduction in P_app_ for encapsulated UA, coupled with the preservation of TEER, highlights the protective role of LNCs in sequestering the compound and preventing the uncontrolled, toxic-driven passage observed with the free drug.

Although the 8TTE mixture is considered a single molecule, we used UPLC–MS to study the behavior of the different isomers. We observed that the feruloyl 8TTE compound present a 3.4- and 3.12-fold (for the free or the encapsulated compounds, respectively) higher permeability than the coumaroyl derivatives, which is enough to pass from low to moderate absorption according to Furtado et al. (2017). This isomer-specific behavior suggests that while LNCs facilitate transport, the inherent lipophilicity and molecular structure of the guest molecule still play a critical role in membrane partitioning. The average permeability data for free 8TTE indicate low intestinal absorption (P_app_ = 6.47 × 10^−7^ ± 0.6^–8^ cm/s), while the average LNC 8TTE formulation data indicate moderate absorption (P_app_ = 1.69 × 10^−6^ ± 2.52^–7^ cm/s). This is the same for UAOPP, with an increase in P_app_ of the formulation to 1.00 × 10^−6^ ± 2.58^–8^ cm/sec. Pure UA is considered moderately absorbed, while LNC UA and the other free drugs can be considered poorly absorbed. It is worthy to notice that UA-tested concentration did not reach saturation, as shown in the study of Jinhua (2019) from 10 to 40 μg/mL. Taken together, these data demonstrate that LNCs constitute suitable carrier systems for improving the intestinal permeability of triterpenic esters with intrinsically low absorption, particularly 8TTE and UAOPP. By shifting these compounds from “low” to “moderate” absorption categories while maintaining epithelial integrity, LNCs demonstrate a clear advantage over free drug administration. LNC encapsulation enhanced P_app_ values while preserving epithelial integrity, as evidenced by transient and fully reversible TEER reductions, indicating that permeability enhancement occurred without compromising tight junction function. The shift of 8TTE and UAOPP from low to moderate absorption categories further supports the ability of LNCs to promote controlled transcellular transport. In contrast, the higher permeability observed for free UA appears to be associated with persistent monolayer disruption rather than true enhancement of epithelial transport, as reflected by the lack of TEER recovery. Encapsulation of UA within LNCs did not improve P_app_ but prevented sustained barrier damage, suggesting a protective effect rather than a permeation-enhancing role. Therefore, although LNCs may not be optimal carriers for increasing UA permeability under the tested conditions, they offer clear advantages for triterpenic esters by enhancing permeability while maintaining epithelial barrier integrity, supporting their relevance as safe and effective oral delivery systems for poorly permeable compounds. The improved permeability observed for triterpenic esters and UA when formulated in LNCs can be attributed to several complementary mechanisms. LNCs, based on a phase-inversion temperature system, enhance oral delivery of lipophilic compounds through improved solubilization and dispersion in the gastrointestinal environment (Amara et al., 2018). Encapsulation within LNCs increases apparent solubility and maintains the compounds in a readily available form at the epithelial surface, thereby sustaining the concentration gradient-driving absorption.

In addition, the presence of non-ionic surfactants (e.g. PEGylated surfactants) within LNCs may induce transient membrane fluidization (potentially inhibiting apical efflux transporters such as P-glycoprotein), thereby enhancing drug partitioning into epithelial cell membranes (Sabri et al., 2022). Furthermore, the nanometric size of LNCs facilitates intimate contact with the intestinal mucosa, promoting transcellular uptake, primarily through endocytosis or transcytosis, rather than paracellular transport (Kaeokhamloed et al., 2021). Overall, the permeability enhancement is likely driven by a combination of improved solubilization, surfactant-mediated membrane fluidization, and transcellular pathways (transcytosis or endocytosis), rather than a single mechanism.

### 3.5 Assessment of the antiparasitic activities

**FIGURE 5:**
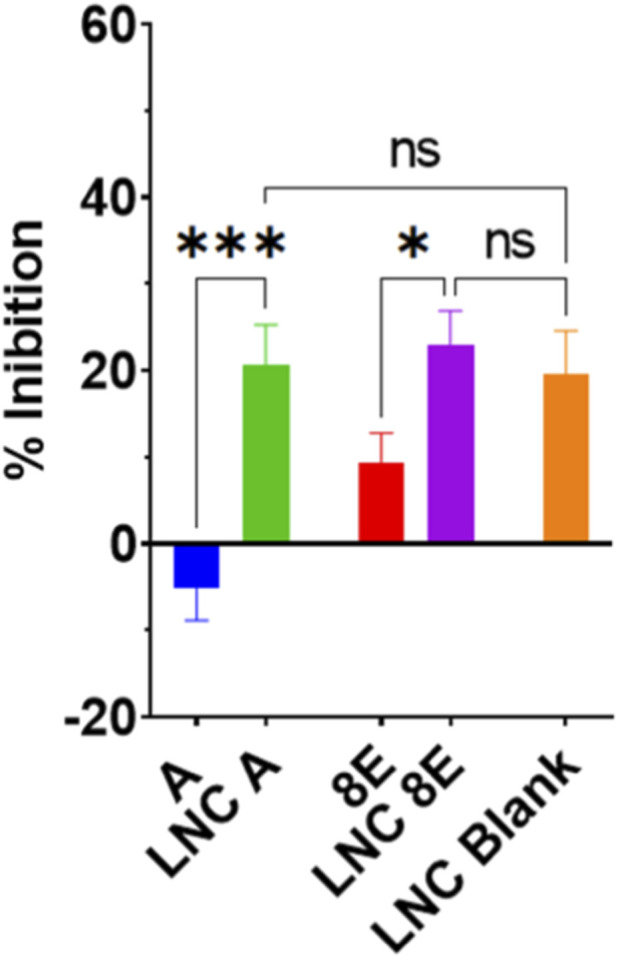
Parasite growth inhibition of *P. falciparum* after incubation with UA (A) and 8TTE (8E) as free drugs or loaded LNCs (mean ± SD; N = 3, n = 6).

**FIGURE 6:**
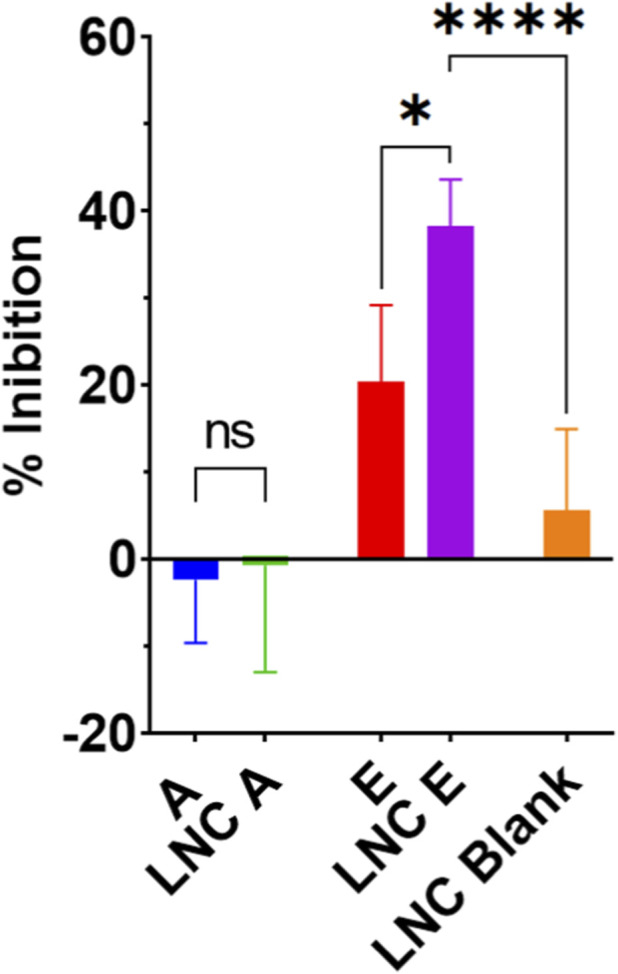
Parasite growth inhibition of *Tbb* after incubation with UA (A) and UAOPP (E) as free drugs or loaded LNCs (mean ± SD; N = 3, n = 6).

The potential cytotoxicity of unloaded LNCs was examined to find the highest no/low toxic concentration to be used to test loaded LNC activity on parasites. Non-toxic unloaded LNC concentrations were observed to be 165 μg/mL and 65 μg/mL on *P. falciparum* and *Tbb*, respectively (Supplementary Figure 4S). Non-toxic concentrations were defined as the highest concentrations of unloaded LNCs that did not induce a statistically significant reduction in parasite viability compared to untreated controls. Antiparasitic data (%) were obtained by incubating the parasites with free drugs and loaded or unloaded (blank) LNCs at concentrations corresponding to the maximum non-toxic concentration of blank LNCs, equivalent to 2.67 µM and 1.06 µM free and loaded UA on *P. falciparum* and *Tbb*, respectively, along with 1.17 µM of 8TTE on *P. falciparum* and 4.3 µM UAOPP on *Tbb*. Concerning the antiplasmodial activity (Figure 5), even if both UA and 8TTE formulations had a significant inhibition improvement (to 20.59% ± 4.71% and 22.93% ± 3.97, respectively) compared to the correspondent free drug, blank LNCs showed an inhibition of 19.57% ± 5.05 compard to both loaded LNCs. This intrinsic antiplasmodial effect of the lipid nanocapsules suggests a mechanistic interaction between the LNC surfactant shell (e.g. Kolliphor HS15) and the plasmodial membrane or potentially an interference with the parasite’s nutrient uptake within the erythrocyte. Specifically, PEGylated surfactants may alter the lipid microdomains of the infected erythrocyte membrane, potentially disrupting the parasite’s specialized transport proteins or internal heme detoxification pathways (Beloqui et al., 2017). For antitrypanosomal activity (Figure 6), parasite viability data were approximately 100% following exposure to free UA or corresponding concentrations of LNC-loaded formulations, showing no effect on *Tbb* at that tested concentration (1.06 µM). It is in accordance with the UA IC_50_ value on *Tbb*, which was reported to be 2.33 µM and is 2.2 times higher than the tested concentration. This explains the inefficacy, and this could not be improved by UA LNC formulation (Schioppa et al., 2021a). This suggests that although LNCs optimize delivery, they cannot overcome the pharmacological limitations of a compound when the dose is significantly below its intrinsic inhibitory threshold. On the contrary, the hemisynthetic ester of UA, UAOPP, was reported to inhibit 20.38% ± 8.80 parasite viability when incubated as free drug at 2.15 µM, and when formulated with LNCs, it showed a significantly higher activity of 38.20% ± 5.41 at the same concentration. This enhanced potency is likely attributable to the LNCs’ ability to increase the biophase concentration of the lipophilic ester at the parasite surface, facilitating its partitioning across the dense glycoprotein coat (pellicle) of the trypanosome. However, as *in vitro* efficacy is significantly dependent on drug delivery dynamics, future *in vivo* investigations are necessary to validate these permeability gains and assess the actual impact of LNC formulation on systemic exposure.

### 3.6 Study limitations

Despite the favorable outcomes of this study, several constraints warrant consideration. First, although the Caco-2 cell monolayer is a gold-standard surrogate for intestinal permeability, it is inherently reductive. It lacks the mucus barrier, the physicochemical complexity of the intraluminal environment (e.g., bile salts and fluctuating pH), and the dynamic mechanical forces of peristalsis. Consequently, although these results provide a mechanistic baseline, they do not account for the potential impact of the intestinal glycocalyx or microbiota-driven degradation on lipid nanocapsules stability. Second, the absence of *in vivo* pharmacokinetic (PK) and biodistribution data precludes a definitive assessment of total systemic exposure (AUC) and peak plasma concentrations (C_max_). *In vitro* transport rates cannot fully predict the impact of hepatic first-pass metabolism or the lymphatic transport route, the latter of which is often significant for highly lipophilic nanocarriers. Finally, although the LNCs preserved epithelial integrity during acute exposure, their chronic safety profile and long-term storage stability (particularly regarding potential drug leakage or particle aggregation) remain to be characterized. Future research will prioritize dose–response *in vivo* efficacy models and comprehensive toxicological profiling to validate the translational potential of these triterpenic ester formulations.

## 4 Conclusion

The aim of this study was to formulate triterpenic esters and UA LNCs and conduct preliminary studies to investigate their potential for their use in the oral delivery of these molecules. In this study, three LNC formulations for C3 or C27 triterpenic esters and UA were prepared and evaluated to improve the antiparasitic activity and cell permeation of triterpenic drugs. The screening of cytotoxic concentration was conducted for cells and parasites for both free and loaded LNC formulations. The antiparasitic potential of the LNC formulation compared to the correspondent free drugs was evaluated and set premises for the enhancement of solubility and permeation of pentacyclic triterpenic esters, although further preclinical studies are required before clinical trials can be conducted. These findings provide a strong foundation for subsequent *in vivo* studies aimed at confirming the therapeutic efficacy and safety of the optimized nanoparticle formulations.

## Data Availability

The original contributions presented in the study are included in the article/Supplementary Material; further inquiries can be directed to the corresponding author.
