# Supplementary material for: Antiparasitic triterpenic ester and ursolic acid lipid nanocapsule design, cytotoxicity, and permeability evaluation for oral delivery
Source: Front Drug Deliv. 2026 Jun 3;6:1846904. doi: 10.3389/fddev.2026.1846904 (PMC13272439; doi:10.3389/fddev.2026.1846904)
Supplement: Supplementary file 1 [file Supplementaryfile1.docx]

Supplementary Material

Antiparasitic Triterpenic Esters and Ursolic Acid

Lipid Nanocapsules Design, Cytotoxicity and Permeability

Evaluation for Oral Delivery

Laura Schioppa, Cecilia Bohns Michalowski, Lucia Mamede, Romano Terrasi, Marie-France Hérent, Giulio Muccioli, Raphaël Fredérick, Michel Frederich, Ana Beloqui, Joelle Quetin-Leclercq^*^

*** Correspondence:** Pr. Joelle Quetin-Leclercq: joelle.leclercq@uclouvain.be

# Preparation of triterpenic esters and ursolic acid lipid nanocapsules (LNC)

#
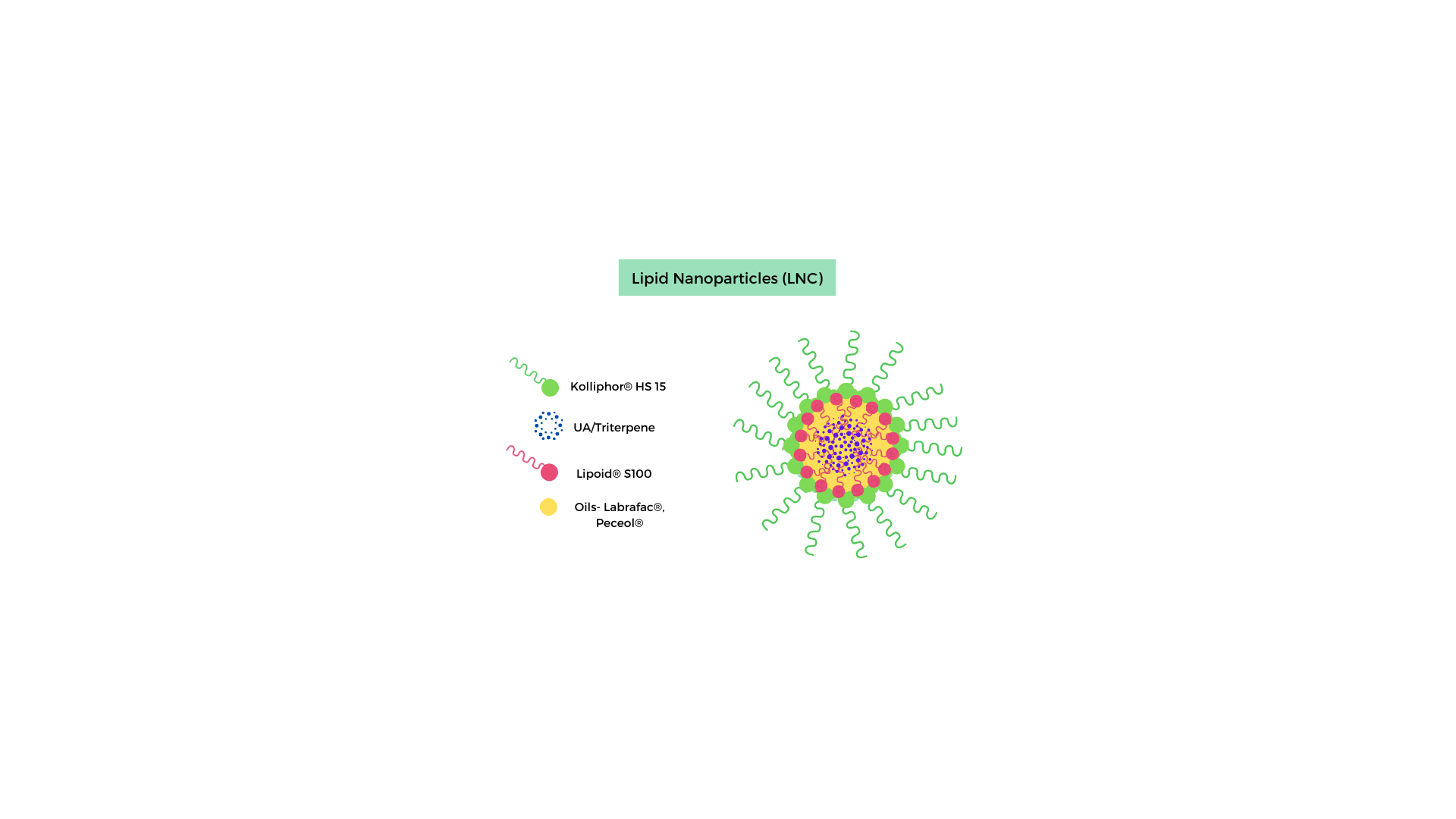


Figure 1S. Schematic representation of grafted lipid nanocapsule (LNCs) for pentacylic triterpenes

# UPLC-MS Method Validation

## MRM chromatograms


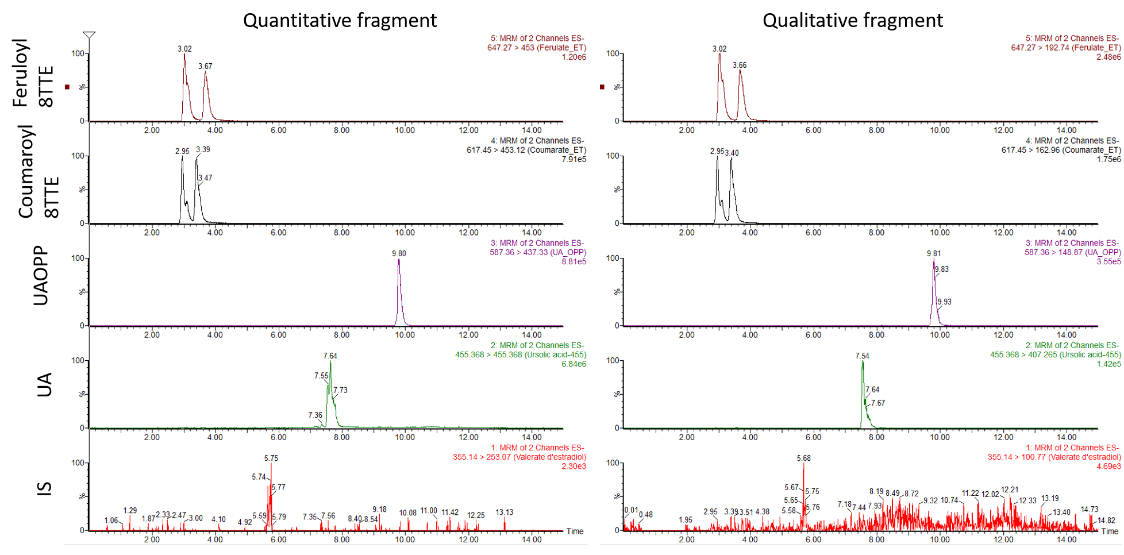


Figure 2S. Representative MRM chromatograms of the quantitative and qualitative fragments of feruloyl 8TTE, coumaroyl 8TTE, UAOPP, and UA and internal standard with the validated UPLC-MS method.

## Selectivity and specificity

## The retention time is 7.55 min for ursolic acid, 9.80 min for UA-3-O-PP, between 2.92 and 3.63 min for 8TTE and 5.77 min for IS. The chromatograms of blank samples from six independent experiments showed no significant peak in either the analyte or the IS MRM in both (quantitative and qualitative) fragments channels, indicating that the method was selective.

## Linearity and lower limit of quantitation and detection

The calibration curve was ranged from 25 to 5000 nM for all compounds. Correlation coefficients of triplicate calibration curves were >0.99 on each validation day. Linearity data for all tested compounds are tabulated below, Table 2S. The limit of detection (LOD) was estimated for all tested compounds to be 2 nM in methanol by the signal/noise method from the European Pharmacopoeia (“European Pharmacopoeia (Ph. Eur.) 10th Edition | EDQM - European Directorate for the Quality of Medicines”, n.d.). The limit of quantification was determined as the smallest tested concentration of the 95% β-expectation tolerance limits remaining inside the ± 20% acceptance limits. It was then set at 25 nM. Linearity parameters, LOD and LOQ are shown in Table 1S.

Table 1S. Validation results obtained for the UPLC-MS quantification method tested triterpenic esters and acid (m=3, n=3, k=3)

| Compound |  | Ursolic acid | UAOPP | 8TTE | | |
| --- | --- | --- | --- | --- | --- | --- |
| Code |  | **(UA)** | **(E/UAOPP)** | **(8E_c_)** | **(8E_f_)** | |
| Linear range | (µM) | 5-0.025 | 5-0.025 | 5-0.025 | | 5-0.025 |
| Response function | Linear regression after square root transformation | | | | | |
| Accuracy | High level (%) | 98.11 | 99.22 | 99.87 | | 100.25 |
|  | Medium level (%) | 103.14 | 100.84 | 100.34 | | 102.93 |
|  | Low level (%) | 103.12 | 98.94 | 99.90 | | 99.76 |
| LOD | (nM) | 2 | 2 | 2 | | 2 |
| LOQ | (nM) | 25 | 25 | 25 | | 25 |
| Inter-day RSD% | High level (%) | 1.79 | 2.05 | 2.52 | | 2.07 |
|  | Medium level (%) | 4.25 | 3.41 | 3.05 | | 2.51 |
|  | Low level (%) | 3.57 | 2.90 | 3.32 | | 1.92 |
| Intra-day RSD % | High level (%) | 3.78 | 3.33 | 4.55 | | 2.43 |
|  | Medium level (%) | 3.90 | 3.57 | 4.33 | | 3.88 |
|  | Low level (%) | 3.29 | 2.54 | 4.17 | | 3.97 |
| Relative Bias % | High level (%) | -1.88 | -0.78 | -0,12 | | 0.25 |
|  | Medium level (%) | 3.15 | 0.84 | 0,34 | | 2.93 |
|  | Low level (%) | 3.12 | -1.06 | -0,09 | | -0.24 |
| Linearity | Slope | 0.98 | 0.99 | 0,99 | | 1,00 |
|  | Intercept | 18.50 | 5.57 | 0,71 | | 8.34 |
|  | R^2^ | 0.99 | 0.99 | 0,99 | | 0.99 |

Low level (%) 75 nM

Medium level (%) 1500 nM

High level (%) 3750 nM

## Precision and accuracy

QC samples at three concentrations were analyzed in six replicates in order to determine the assay accuracy and precision. As shown in Table 1S, the intra- and inter-day precisions were less than 4.55% and 4.25%, and the accuracy was within ±3.15% showing a good precision and accuracy of the method.

## Uncertainty of measurement

# To characterize values dispersion during routine analysis, the uncertainty of measurement has been determined (2,3). The expanded uncertainty is calculated by applying a coverage factor of k=2 (corresponding to a 95% confidence interval around the estimated result). The maximum relative expanded uncertainty of the mixture of 8TTE is 10.2 % while for UA-3-O-PP is 7.6% and 8.4% for UA, all inside the ± 20% acceptance limits. All estimated values for each validation standard concentration level are summarized in Table 2S.

Table 2S. Validation results obtained for the UPLC-MS quantification method tested triterpenic esters and acid. CL: concentration level E: UAOPP; 8Ec: 8TTE coumaroyl; 8Ef: 8TTE feruloyl

| CL | Uncertainty  (nM) | | | | Expanded uncertainty  (nM) | | | | Relative expanded uncertainty  (%) | | | |
| --- | --- | --- | --- | --- | --- | --- | --- | --- | --- | --- | --- | --- |
|  | UA | E | 8E_c_ | 8E_f_ | UA | E | 8E_c_ | 8E_f_ | A | E | 8E_c_ | 8E_f_ |
| H | 157.5 | 138.3 | 191.6 | 98.8 | 315.0 | 276.7 | 383.2 | 197.6 | 8.4 | 7.4 | 10.2 | 5.3 |
| M | 62.4 | 57.4 | 72.1 | 66.6 | 124.8 | 114.7 | 144.1 | 133.3 | 8.3 | 7.6 | 9.6 | 8.9 |
| L | 2.6 | 1.9 | 3.4 | 3.4 | 5.3 | 3.8 | 6.8 | 6.7 | 7.0 | 5.1 | 9.1 | 9.0 |

L: Low level (%) 75 nM; M: Medium level (%) 1500 nM; H: High level (%) 3750 nM

## Stability

Results of the stability tests were summarized in Table 4S. Repeatability and precision (RB, RSD) were all within the ±15% limits as described by EMA guidelines (4). All tested compounds were stable after being stored at -20 °C and -80 °C for one month.

Table 3S. Stability validation results obtained for the UPLC-MS quantification method tested triterpenic esters and acid. CL: concentration level; 8TTEc: 8TTE coumaroyl; 8TTEf: 8TTE feruoyl.

| **CL** | **Compound** | **Stability -20°C, 1 month** | | | **Stability -80°C, 1 month** | | |
| --- | --- | --- | --- | --- | --- | --- | --- |
|  |  | RB (%) | RDS (%) | iRDS (%) | RB (%) | RDS (%) | . iRDS (%) |
| High | UA | -1,88 | 1.79 | 3.78 | -0.26 | 2.40 | 6.65 |
|  | UAOPP | 4.12 | 5.38 | 4.79 | 2.93 | 2.20 | 2.02 |
|  | 8TTE_c_ | 3.74 | 2.49 | 2.03 | 1.30 | 6.29 | 5.89 |
|  | 8TTE_f_ | 1.91 | 2.81 | 2.29 | 3.04 | 3.08 | 3.39 |
| Low | UA | -3.75 | 0.20 | 6.38 | -1.90 | 3.63 | 6.03 |
|  | UAOPP | 3.46 | 1.72 | 3.42 | -1.61 | 5.46 | 4.61 |
|  | 8TTE_c_ | 1.30 | 6.29 | 5.89 | -1.73 | 4.19 | 5.56 |
|  | 8TTE_f_ | -2.10 | 1.85 | 1.51 | -0.02 | 3.99 | 4.17 |

RB: relative bias; RSD: relative standard deviation; iRSD= intermediate relative standard deviation; Low level (%) 75 nM; High level (%) 3750 nM

# Caco-2 cell viability study


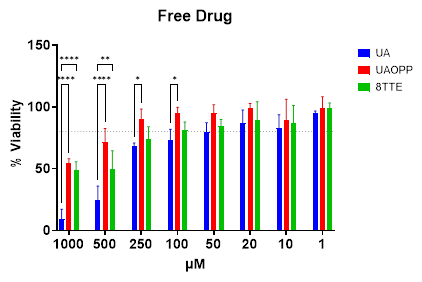


Figure 3S. Cell viability of Caco-2 cells at different concentrations (1 µM to 1000 µM) of UA, UAOPP, 8TTE (mean ± SD; N =3, n=3) (ANOVA, Dunnett’s test compared to UA at the same concentration)

# Cytotoxic assessment of the LNCs formulations on parasites

Figure 4S. Cytotoxicity of blank lipid nanocapsules (LNC) on (a) *Plasmodium falciparum* (*Pf*) (192–0.09 µg/mL) and (b) *Trypanosoma brucei brucei* (*Tbb)* (100–0.05 µg/mL). Cell viability is expressed as a percentage of the control. Data are presented as mean ± SD (N = 3, n = 3).

# Figure Legends

[Figure 1S. Schematic representation of grafted lipid nanocapsule (LNCs) for pentacylic triterpenes 1](#_Toc219784538)

[Figure 2S. Representative MRM chromatograms of the quantitative and qualitative fragments of feruloyl 8TTE, coumaroyl 8TTE, UAOPP, and UA and internal standard with the validated UPLC-MS method. 2](#_Toc219784539)

[Figure 3S. Cell viability of Caco-2 cells at different concentrations (1 µM to 1000 µM) of UA, UAOPP, 8TTE (mean ± SD; N =3, n=3) (ANOVA, Dunnett’s test compared to UA at the same concentration) 5](#_Toc219784540)

[Figure 4S. Cytotoxicity of blank lipid nanocapsules (LNC) on (a) *Plasmodium falciparum* (*Pf*) (192–0.09 µg/mL) and (b) *Trypanosoma brucei brucei* (*Tbb)* (100–0.05 µg/mL). Cell viability is expressed as a percentage of the control. Data are presented as mean ± SD (N = 3, n = 3). 5](#_Toc219784541)

# Table Legends

[Table 1S. Validation results obtained for the UPLC-MS quantification method tested triterpenic esters and acid (m=3, n=3, k=3) 3](#_Toc205537202)

[Table 2S. Validation results obtained for the UPLC-MS quantification method tested triterpenic esters and acid. CL: concentration level E: UAOPP; 8Ec: 8TTE coumaroyl; 8Ef: 8TTE feruloyl 4](#_Toc205537203)

[Table 3S. Stability validation results obtained for the UPLC-MS quantification method tested triterpenic esters and acid. CL: concentration level; 8TTEc: 8TTE coumaroyl; 8TTEf: 8TTE feruoyl. 4](#_Toc205537204)

# References

1. European Pharmacopoeia (Ph. Eur.) 10th Edition | EDQM - European Directorate for the Quality of Medicines. https://www.edqm.eu/en/european-pharmacopoeia-ph-eur-10th-edition [Accessed March 15, 2021]

2. Rozet E, Marini RD, Ziemons E, Boulanger B, Hubert P. Advances in validation, risk and uncertainty assessment of bioanalytical methods. *J Pharm Biomed Anal* (2011) 55:848–858. doi: 10.1016/j.jpba.2010.12.018

3. Lautié E, Rozet E, Hubert P, Quetin Leclercq J. Quantification of rotenone in seeds of different species of yam bean (Pachyrhizus sp.) by a SPE HPLC-UV method. *Food Chem* (2012) 131:1531–1538. doi: 10.1016/j.foodchem.2011.09.125

4. Medicines Agency E. 2** Committee for Medicinal Products for Human Use (CHMP) Guideline on bioanalytical method validation. (1922). www.ema.europa.eu/contact [Accessed March 15, 2021]

**
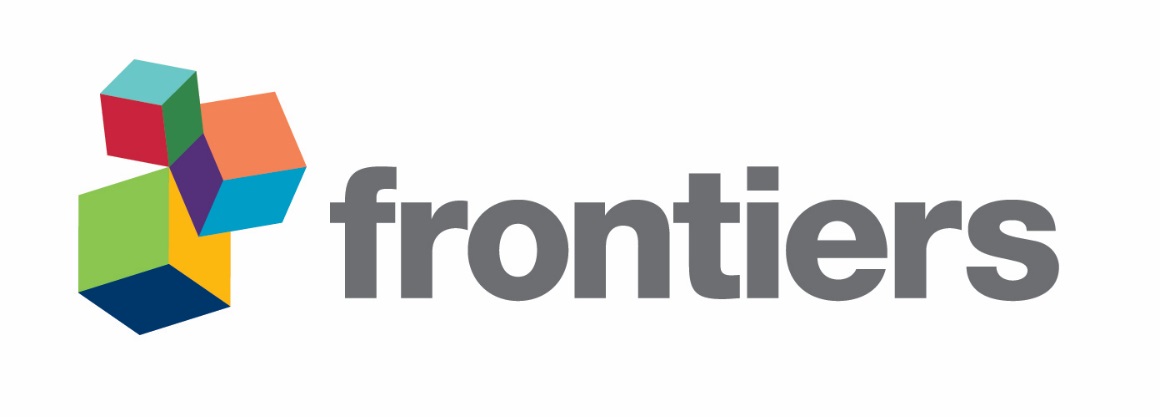
**
